# Increased VLCFA-lipids and ELOVL4 underlie neurodegeneration in frontotemporal dementia

**DOI:** 10.1038/s41598-021-00870-x

**Published:** 2021-11-01

**Authors:** Ying He, Katherine Phan, Surabhi Bhatia, Russell Pickford, YuHong Fu, Yue Yang, John R. Hodges, Olivier Piguet, Glenda M. Halliday, Woojin Scott Kim

**Affiliations:** 1 Brain and Mind Centre and School of Medical Sciences, The University of Sydney, Camperdown, Sydney, NSW 2050 Australia; 2 Bioanalytical Mass Spectrometry Facility, University of New South Wales, Sydney, NSW Australia; 3 Brain and Mind Centre and School of Psychology, The University of Sydney, Sydney, NSW Australia; 4 Neuroscience Research Australia, Sydney, NSW Australia; 5 School of Medical Sciences, University of New South Wales, Sydney, NSW Australia

**Keywords:** Cellular neuroscience, Lipidomics

## Abstract

Rare, yet biologically critical, lipids that contain very long chain fatty acids (VLCFA-lipids) are synthesized in the brain by the enzyme ELOVL4. High levels of VLCFA-lipids are toxic to cells and excess VLCFA-lipids are actively removed by ABCD1 in an ATP-dependent manner. Virtually nothing is known about the impact of VLCFA-lipids in neurodegenerative diseases. Here, we investigated the possible role of VLCFA-lipids in frontotemporal dementia (FTD), which is a leading cause of younger-onset dementia. Using quantitative discovery lipidomics, we identified three VLCFA-lipid species that were significantly increased in FTD brain compared to controls, with strong correlations with ELOVL4. Increases in ELOVL4 expression correlated with significant decreases in the membrane-bound synaptophysin in FTD brain. Furthermore, increases in ABCD1 expression correlated with increases in VLCFA-lipids. We uncovered a new pathomechanism that is pertinent to understanding the pathogenesis of FTD.

## Introduction

Frontotemporal dementia (FTD) is a leading cause of younger-onset dementia arising primarily from neuronal dysfunction and degeneration. The etiology of FTD in most cases is largely unknown, although repeat expansions in the *C9ORF72* gene are a common genetic cause. The brain is enriched in lipids, and alterations in lipids in FTD significantly contribute to mitochondrial dysfunction, inflammation and oxidative stress^1^. There has been no previous studies on brain-specific lipids in FTD, particularly those implicated in neurodegeneration—lipids that contain very long chain fatty acids (VLCFA-lipids). VLCFA-lipids contain > 26 carbon atoms in the fatty acid chain. They are normally extremely low in abundance (< 1% of total lipids)^2,3^ and are synthesized by an enzyme called ELOngation of Very Long chain fatty acids 4 (ELOVL4)^4^. Both VLCFA-lipids and ELOVL4 are present in only brain, retina, skin, testes and meibomian gland^2–4^. Very long chain fatty acids render VLCFA-lipids extremely hydrophobic with increasing hydrophobicity with increasing fatty acid chain length^5^.

VLCFA-lipids are localized to cellular membranes, where they play integral roles in membrane function. Alterations in VLCFA-lipids distort the physicochemical properties of membranes and perturb the function/activity of proteins embedded in membranes, resulting in cellular dysfunction and cell death^6^. VLCFA-lipids localized in synaptic vesicles regulate the kinetics of presynaptic neurotransmitter release^7,8^. High levels of VLCFA-lipids are toxic to cells and excess VLCFA-lipids are actively removed by ATP-binding cassette subgroup D member 1 (ABCD1) localized on peroxisomes, where they are broken down by the process of β-oxidation^9^. Mutations in the *ABCD1* gene cause adrenoleukodystrophy, which is characterized by a buildup of VLCFA-lipids in the brain, progressive neurodegeneration, dementia, demyelination and difficulty in speaking and listening^10^.

Despite the importance of VLCFA-lipids and ELOVL4 in neurological processes, very little is known about VLCFA-lipids/ELOVL4 in neurodegenerative diseases. Here, we utilized quantitative discovery lipidomics to identify VLCFA-lipids that are altered in FTD brain and investigate the impact of VLCFA-lipids on membrane function.

## Results

### Increased levels of VLCFA-lipids in FTD brain

**Figure 1:**
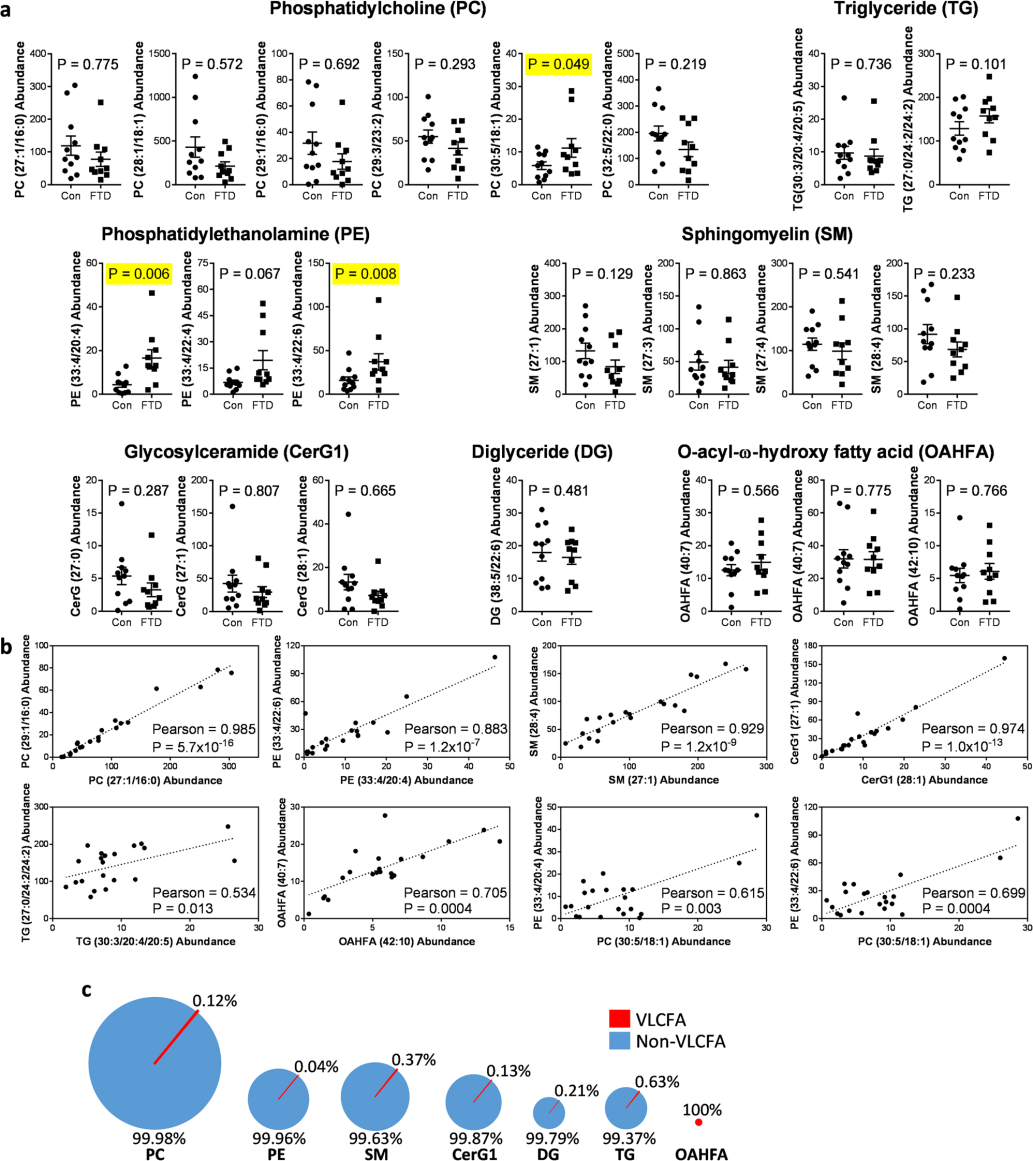
VLCFA-lipids are increased in FTD brain. (**a**) We identified 22 VLCFA-lipid species in the human brain as measured by quantitative discovery lipidomics LC/MS. Multivariate analysis (general linear model), co-varying for age and sex, was used to determine lipids significantly altered in FTD (n = 10) compared to controls (n = 11) and are highlighted in yellow. Relative abundance of lipids was obtained from LC/MS peak areas normalized to internal standards. Data represent mean and SE as error bars. (**b**) Pearson correlation (combined of both FTD cases and controls) was used to determine the relationship between VLCFA-lipid species. (**c**) Distribution of VLCFA-lipids compared to non-VLCFA-lipids for each lipid class.

The level and distribution of VLCFA-lipids in FTD brain is unknown. Here, we used liquid chromatography/mass spectrometry (LC/MS) technology and LipidSearch software to identify and measure VLCFA-lipids, as well as non-VLCFA-lipids, in the superior frontal cortex of FTD and control brain. We identified 22 individual VLCFA-lipid species in seven classes of lipids—6 phosphatidylcholine (PC), 3 phosphatidylethanolamine (PE), 4 sphingomyelin (SM), 3 glycosylceramide (CerG1), 1 diglyceride (DG), 2 triglyceride (TG) and 3 o-acyl-ω-hydroxy fatty acid (OAHFA) (Fig. 1a). Out of the 22 species, only three were significantly increased in FTD compared to controls—PC(30:5/18:1), PE(33:4/20:4) and PE(33:4/22:6) (Fig. 1a). We assessed the correlation (combined of both FTD cases and controls) between the species from the same class and also between the species from different classes. In each case, there was a strong correlation between the species, including the three species that were increased in FTD, and several examples are shown for illustration purpose (Fig. 1b). The strong correlation among the species, despite the fact that the lipids are structurally different and are synthesized under independent pathways, suggests the importance of VLCFA-lipids in brain function. We also assessed the distribution of VLCFA-lipids in each class and found that VLCFA-lipids were extremely low in abundance (i.e. < 1%), except for OAHFA (Fig. 1c). OAHFA is structurally different to other lipid classes in that it is composed entirely of only fatty acid. These results strongly suggest that an increase in VLCFA-lipids in the brain is associated with the neurodegeneration observed in FTD.

### Increased ELOVL4 protein in FTD brain

**Figure 2:**
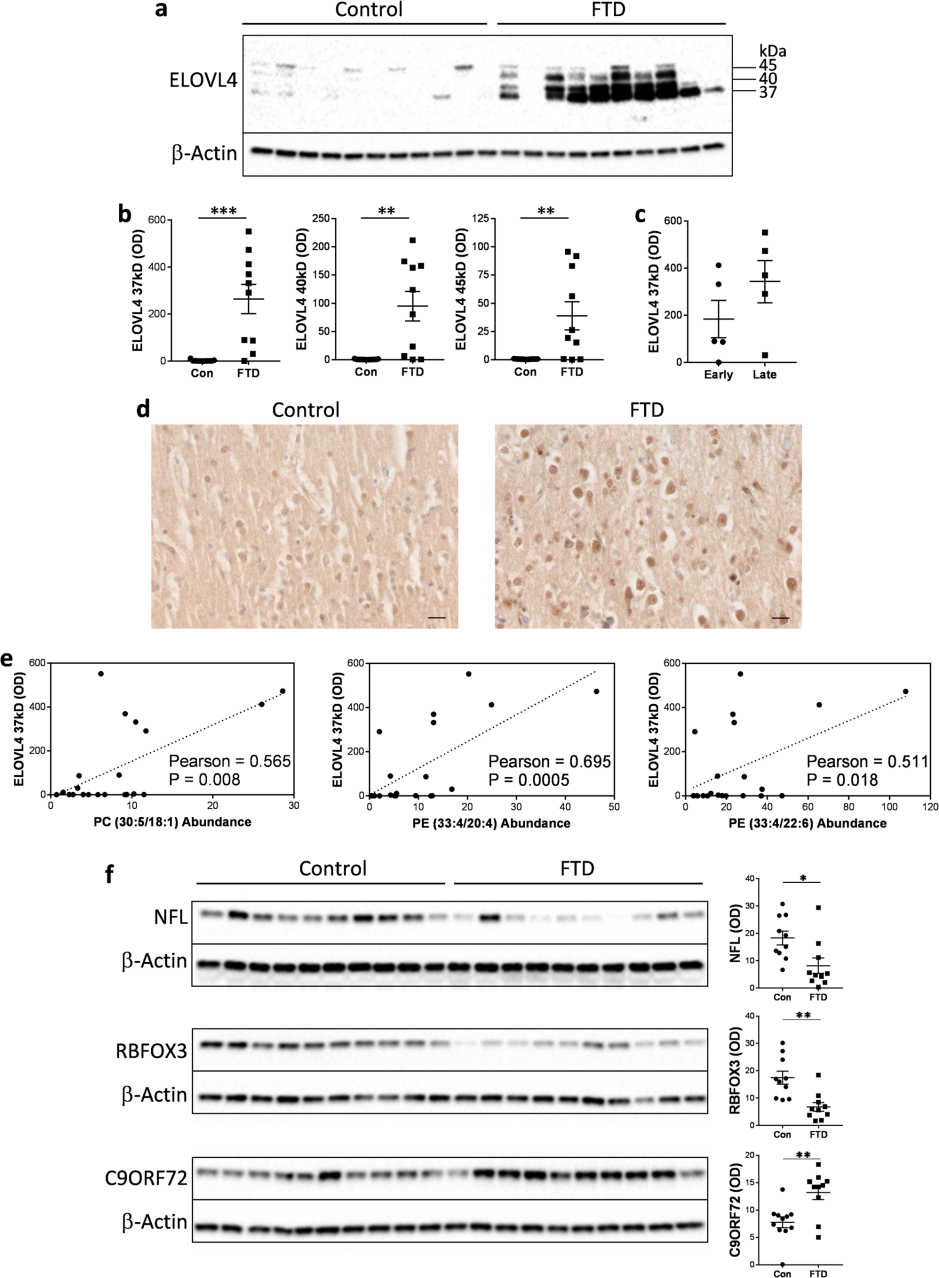
ELOVL4 protein is increased in FTD brain. (**a**) ELOVL4 protein was significantly increased in superior frontal cortex of FTD brain (n = 10) compared to controls (n = 11) as measured by western blotting. (**b**) Optical density (OD) measurements of the ELOVL4 protein bands normalized to β-actin. Multivariate analysis (general liner model), co-varying for age and sex, was used to determine significance between the two groups. (**c**) A comparison of the 37-kDa ELOVL4 protein (OD) levels in early-stage (n = 5) and late-stage (n = 5) FTD groups. (**d**) A representative example of (non-quantitative) immunohistochemical staining of ELOVL4 in superior frontal cortex of FTD and control brain; scale bar = 50 µm. (**e**) The 37-kDa ELOVL4 protein (OD) levels correlated significantly with the levels of all three VLCFA-lipids elevated in FTD brain as determined by Pearson correlation coefficient. (**f**) Protein expression and OD measurement, normalized to β-actin, of neurofilament light (NFL), RNA binding fox-1 homolog 3 (RBFOX3) and C9ORF72 in superior frontal cortex of FTD (n = 10) and controls (n = 10) brain, and significance determined by multivariate analysis (general liner model), co-varying for age and sex. Data represent mean and SE as error bars, *P < 0.05, **P < 0.005, ***P < 0.001.

To determine the cause of the increase in the three VLCFA-lipids in FTD brain, we assessed the expression of ELOVL4, which is solely responsible for VLCFA synthesis. The 37-kDa ELOVL4 protein was dramatically increased in FTD compared to controls (Fig. 2a,b). Two larger proteins at 40 and 45 kD (possibly glycosylated forms) were also increased in FTD (Fig. 2a,b). When the FTD samples were split into early-stage and late-stage groups, ELOVL4 expression was higher in the late-stage group (Fig. 2c), suggesting that ELOVL4 expression is associated with disease progression. Assessment of ELOVL4 by immunohistochemistry (Fig. 2d) confirmed the expression of ELOVL4 in neurons as well as glia^11^. Importantly, the increases in ELOVL4 expression, as measured by western blotting, correlated significantly with all three VLCFA-lipids elevated in FTD brain (Fig. 2e). We also assessed three other proteins that are important to neuronal function—neurofilament light (NFL), RNA binding fox-1 homolog 3 (RBFOX3; also called neuronal nuclei (NeuN)) and C9ORF72. NFL and RBFOX3 are quantitative markers for neurons, and C9ORF72 is a gauge for neuronal survival activity^12,13^. Both NFL and RBFOX3 were decreased, whereas C9ORF72 was increased, in FTD compared to controls (Fig. 2f). These results suggest that ELOVL4 and its products, VLCFA-lipids, are particularly enriched in remaining active neurons in regions of degeneration in FTD.

### Increases in VLCFA-lipids cause synaptophysin dysregulation

**Figure 3:**
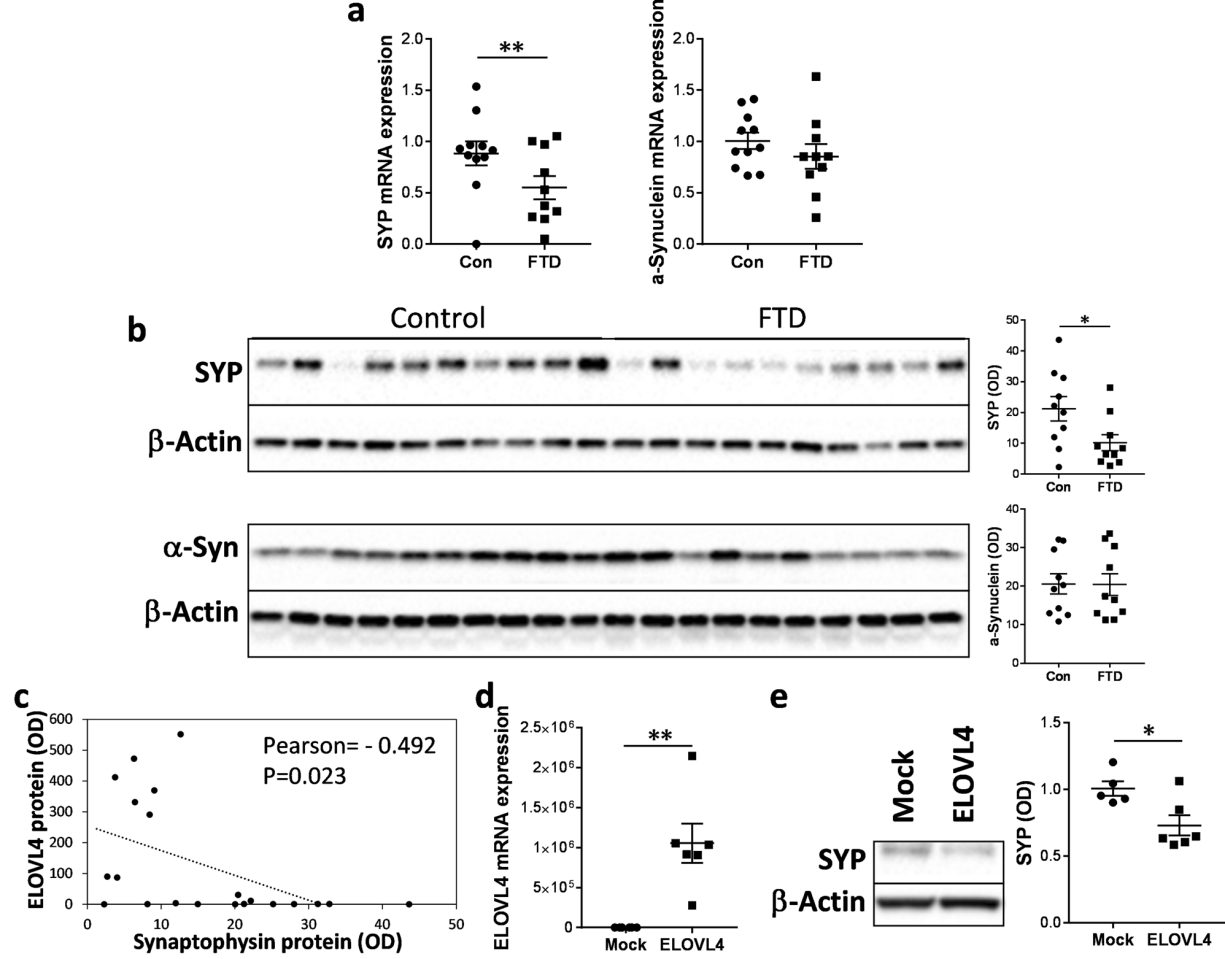
Dysregulation of synaptophysin in FTD brain. (**a**) mRNA expression of synaptophysin (SYP) and α-synuclein, normalized to the geometric mean of β-actin, GAPDH and PPIA, in superior frontal cortex of FTD brain (n = 10) and controls (n = 11) as measured by qPCR. Multivariate analysis (general liner model), co-varying for age and sex, was used to determine significance between the two groups. (**b**) Protein expression of SYP and α-synuclein, normalized to β-actin, in superior frontal cortex of FTD brain (n = 10) and controls (n = 10) as measured by western blotting and optical density (OD) measurements of the protein bands. Multivariate analysis (general liner model), co-varying for age and sex, was used to determine significance between the two groups. (**c**) SYP protein (OD) was inversely correlated with the 37-kDa ELOVL4 protein (OD). (**d**) SH-SY5Y neuronal cells were transfected with ELOVL4 cDNA plasmid or empty vector (mock control) (n = 6 each; 2 experimental repeats), and ELOVL4 mRNA expression, normalized to the geometric mean of β-actin, GAPDH and PPIA, measured by qPCR. Student’s *t*-test was used to determine significance between the two groups. (**e**) SYP protein expression in the transfected SH-SY5Y neuronal cells as assessed by western blotting and OD measurements of the protein bands normalized to β-actin. Student’s *t*-test was used to determine significance between the two groups. Data represent mean and SE as error bars, *P < 0.05, **P < 0.005.

VLCFA-lipids and ELOVL4 are associated with significant synaptic vesicle dysfunction in neurological and macular conditions^8,14,15^. Here, we measured the synaptic vesicle membrane protein synaptophysin (SYP) in the superior frontal cortex of FTD and controls, the same tissues that were used for VLCFA-lipid and ELOVL4 analyses. SYP is the most abundant synaptic vesicle protein and is a marker for synaptic vesicle integrity. We also measured the presynaptic protein α-synuclein. Both mRNA and protein expression of SYP, but not α-synuclein, were significantly decreased in FTD compared to controls (Fig. 3a,b). Also, SYP levels were inversely correlated with ELOVL4 levels (Fig. 3c). To verify this inverse relationship, we then overexpressed ELOVL4 in SH-SY5Y neuronal cells (modelling FTD brain tissue; Fig. 3d) and measured the effect on SYP expression. Increases in ELOVL4 expression correlated with significant decreases in SYP expression (Fig. 3e), corroborating the brain data. When put together, these results suggest that high levels of VLCFA-lipids detrimentally impact on SYP levels in FTD brain.

### Upregulation of ABCD1 in response to high levels of VLCFA-lipids

**Figure 4:**
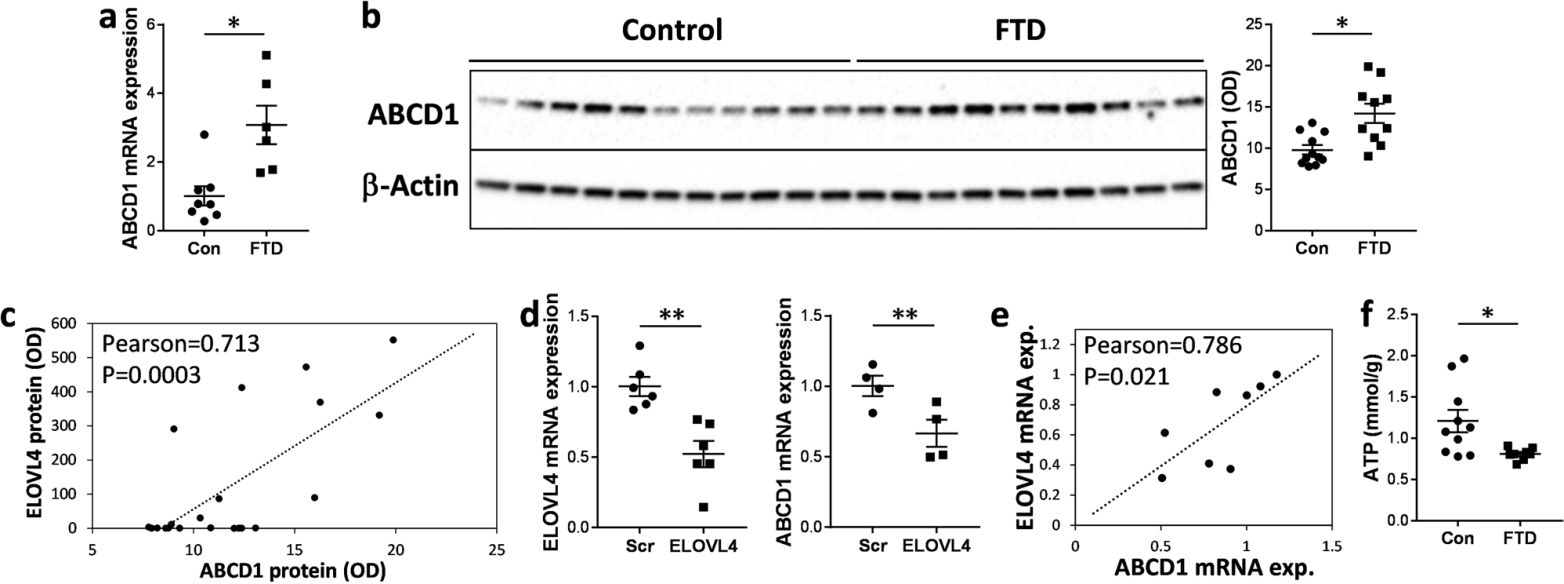
Upregulation of ABCD1 in response to high levels of VLCFA-lipids. (**a**) mRNA expression of ABCD1, normalized to the geometric mean of β-actin, GAPDH and PPIA, in superior frontal cortex of FTD brain (n = 10) and controls (n = 11) as measured by qPCR. Multivariate analysis (general liner model), co-varying for age and sex, was used to determine significance between the two groups. (**b**) Protein expression of ABCD1 in superior frontal cortex of FTD brain (n = 10) and controls (n = 11) as measured by western blotting and optical density (OD) measurement of the protein bands normalized to β-actin. Multivariate analysis (general liner model), co-varying for age and sex, was used to determine significance between the two groups. (**c**) ABCD1 protein (OD) correlated significantly with the 37-kDa ELOVL4 protein (OD) as determined by Pearson correlation coefficient. (**d**) SH-SY5Y neuronal cells were knocked down with ELOVL4 siRNA or scramble control (Scr) siRNA (n = 4 each; 3 experimental repeats), and ELOVL4 and ABCD1 mRNA expression, normalized to the geometric mean of β-actin, GAPDH and PPIA, assessed by qPCR. Student’s *t*-test was used to determine significance between the two groups. (**e**) ABCD1 mRNA expression correlated significantly with ELOVL4 mRNA expression in the SH-SY5Y neuronal cells as determined by Pearson correlation coefficient. (**f**) ATP levels were significantly decreased in FTD brain (n = 10) compared to controls (n = 11). Multivariate analysis (general liner model), co-varying for age and sex, was used to determine significance between the two groups. Data represent mean and SE as error bars, *P < 0.05, **P < 0.005.

High levels of VLCFA-lipids are toxic to cells and excess VLCFA-lipids are actively removed by ABCD1 in an ATP-dependent manner^16^. Here we measured the expression of ABCD1 in the superior frontal cortex of FTD and controls. Both mRNA and protein expression of ABCD1 were significantly upregulated in FTD (Fig. 4a,b). Importantly, there was a strong correlation between the levels of ABCD1 and ELOVL4 (Fig. 4c). To verify these results, we altered the expression of ELOVL4 in SH-SY5Y neuronal cells by knockdown and measured ABCD1 expression. We found that the expression of ABCD1 was significantly reduced with decreases in ELOVL4 (Fig. 4d), and that ABCD1 levels correlated strongly with ELOVL4 levels (Fig. 4e). Since the function of ABCD1 (an ATP-binding cassette transporter) is dependent on supply of energy in the form of ATP, we also measured ATP levels in the same tissue samples. We found that ATP levels were significantly lower in FTD compared to controls (Fig. 4f), which is consistent with the fact that mitochondria (that generates ATP) are dysfunctional in FTD^17^. These results suggest that ABCD1 expression is upregulated likely in response to high levels of VLCFA-lipids. However, its ability to remove excess VLCFA-lipids could be compromised because of the reduction in ATP levels in FTD brain.

### Detection of VLCFA-lipids in FTD blood serum

**Figure 5:**
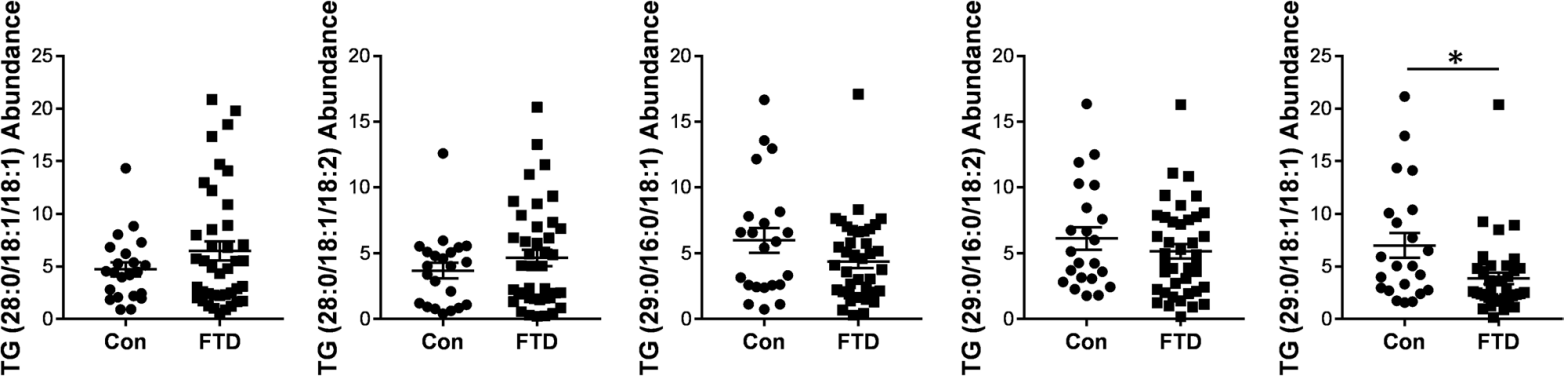
Assessment of VLCFA-lipids in FTD serum. Quantitative discovery lipidomics LC/MS detected five VLCFA-TG species in the human serum, of which one was significantly decreased in FTD (n = 40) compared to controls (n = 22). Relative abundance of lipids was obtained from LC/MS peak areas normalized to internal standards. Multivariate analysis (general liner model), co-varying for age and sex, was used to determine significance between the two groups. Data represent mean and SE as error bars, *P < 0.05.

In addition to understanding the role of VLCFA-lipids in FTD pathogenesis, we were interested to know if VLCFA-lipids could be detected in the blood serum. For this study, we used serum collected from a cohort of sporadic FTD patients used in our previous studies^1^. We undertook a comprehensive analysis of serum lipids using quantitative discovery lipidomics LC/MS, the same technique used to analyze the brain lipids. We found only five VLCFA-lipid species in the serum. They were all triglyceride, four of them were unchanged and one was decreased in FTD compared to controls (Fig. 5). No VLCFA-PC or VLCFA-PE species were detected in the serum. Finally, we attempted to measure ELOVL4 in the same serum by western blotting and ELISA, but found no trace of it.

## Discussion

Recent developments in mass spectrometry and analytical software have enabled discovery of low abundant yet biologically important lipids present in tissues and biofluids. One such group is VLCFA-lipids. Extremely low levels of VLCFA-lipids are present in only certain tissues, including the brain, as confirmed by our findings. They are synthesized solely by the enzyme ELOVL4 and are localized to cellular membranes, where they play integral roles in membrane function. VLCFA-lipids are highly hydrophobic and “bulky” because of their long tails, and they affect the physicochemical properties of membranes, such as fluidity, permeability and lipid microdomain^14^. Alterations in VLCFA-lipids impact on proteins embedded in membranes, as demonstrated by the changes in SYP level in FTD brain. The pathological relevance of VLCFA-lipids in brain function has been mostly studied in the context of neurological or macular diseases, however, emerging data show that VLCFA-lipids are also altered in neurodegenerative diseases. For example, VLCFA-lipids are increased in affected cortical regions of Alzheimer’s disease (AD) brain^18^. Interestingly, all five lipids that were increased in cerebral spinal fluid of amyotrophic lateral sclerosis (ALS) were VLCFA-lipids^19^. There are a number of physiological traits that overlap between FTD and AD^20,21^, and FTD and ALS are considered to be on the same disease spectrum^22^.

Virtually nothing is known about VLCFA-lipids in FTD. Here, we investigated the possible impact of VLCFA-lipids in FTD brain, particularly their impact on the membrane-encased synaptic vesicles in neurons. We uncovered three VLCFA-lipid species, i.e. one VLCFA-PC and two VLCFA-PE, that were significantly increased in the superior frontal cortex of FTD brain, a region of the brain affected by FTD. All three VLCFA-lipids were strongly correlated with ELOVL4 and increased with disease severity and neurodegeneration. VLCFA-PC species were previously shown to be present in the human brain and retina^23,24^. Our discovery of VLCFA-PE species in the human brain is novel. PC is the most abundant lipid in mammalian cellular membranes, followed by PE^25,26^. In synaptic vesicle membranes, PC makes up 40% and PE 32% of the total lipid^27^, and they are present predominantly in the inner leaflets^26^ that have the greatest curvatures and flexibilities.

Since VLCFA-lipids were increased in FTD brain and the fact that ABCD1 is responsible for removing excess VLCFA-lipids, we were interested in whether ABCD1 expression is altered in FTD brain. We found that both mRNA and protein expression of ABCD1 were increased in FTD brain, indicating that the expression of ABCD1 was upregulated in response to the high levels of VLCFA-lipids. ABCD1 is a member of the ATP-binding cassette (ABC) transporter family that requires ATP to transport substrates across membranes (i.e. an active process). ABCD1 specifically transports VLCFA-lipids across peroxisome membranes, where VLCFA-lipids are catabolized by the process of β-oxidization^9^. We found that ATP levels were significantly decreased in FTD brain, which is consistent with mitochondrial dysfunction in FTD brain^17^. Neurons generate ATP primarily via mitochondrial oxidative phosphorylation using glucose as the predominant substrate^28^, and reduced cellular glucose consumption is one of the first indices of FTD neurodegeneration. Without adequate supply of ATP, ABCD1 cannot function properly, i.e. remove excess VLCFA-lipids, irrespective of its expression level. High levels of VLCFA-lipids are toxic to neurons, as well as other cells, as unequivocally demonstrated in adrenoleukodystrophy. Adrenoleukodystrophy is caused by mutations in *ABCD1* that result in a buildup of VLCFA-lipids in neural tissues, including the brain^29^. Adrenoleukodystrophy is characterized by a number of neurological symptoms, including neuropathy, emotional instability, hyperactivity, altered behavior, and dementia, some of which overlap with those of FTD. Attempts were made to treat adrenoleukodystrophy patients by restricting the dietary intake of VLCFA-lipids. This was however unsuccessful because of the fact that much of the body’s VLCFA-lipids are synthesized endogenously^30^.

Gene therapy has also been trialed to treat adrenoleukodystrophy patients. Insertion of a lentiviral vector carrying the wild type *ABCD1* gene has resulted in improving some of the neurological symptoms in two reported cases^31^. No studies have been reported on reducing VLCFA-lipid synthesis by inhibition of the ELOVL4 enzyme. A potential problem of such inhibition is that too low a level can also cause severe neurological abnormalities, as well as other physiological dysfunction, as clearly demonstrated by mutations in *ELOVL4*. Heterozygous *ELOVL4* mutations cause autosomal dominant spinocerebellar ataxia^32^ or macular dystrophy^33^, whereas homozygous *ELOVL4* mutations cause intellectual disability, spastic quadriplegia and ichthyosis^34^. It is clear from both human and animal mutation studies, loss of VLCFA-lipids causes significant biological dysfunction within the tissues (i.e. brain, retina, skin, testes and meibomian gland). From these observations it appears that regulation of VLCFA-lipid levels in specific tissues is extremely important, as too high or too low levels of VLCFA-lipids result in profound phenotypic changes.

Nonetheless, since some of the symptoms of adrenoleukodystrophy overlap with those of FTD it would be interesting to consider the various adrenoleukodystrophy treatment strategies for possible application in FTD therapy. Controlling the dietary intake of VLCFA-lipids in FTD would unlikely to impact on VLCFA-lipid levels in the brain because of the blood brain barrier that would prevent large lipid species crossing either direction. This is consistent with our findings that VLCFA-PC and VLCFA-PE present in the brain were not detected in the serum. This is also consistent with the findings that dietary therapy designed to reduce VLCFA-lipid levels had no effect on halting the neurologic progression or improving the endocrine dysfunction in patients with adrenoleukodystrophy and in symptomatic heterozygous women^35,36^.

Interestingly, both VLCFA-PE species, PE(33:4/20:4) and PE(33:4/22:6), that were increased in FTD brain were odd-numbered chain fatty acids (OCFA); both contain 33 carbon atoms in one of the fatty acid chains. In mammals, only a very small proportion of fatty acids are OCFA. However, the proportion of OCFA is relatively high in the brain, suggesting a greater physiologic relevance of OCFA in the brain^37^. OCFA are synthesized by elongation systems^38^ or derived from even-numbered fatty acids^39^. The derivation process targets 2-hydroxylated fatty acids, which are essential components of glycosphingolipids present in the brain^40^. The increase, even if only small overall, of VLCFA-lipids into highly curved and flexible membrane leaflets would alter these membrane characteristics and function that would impact on membrane-bound proteins. We found that membrane-bound SYP was specifically reduced in remaining synapses in FTD brain, which is consistent with the fact that loss of SYP increases synaptic vesicle release^41^. The level of α-synuclein was unchanged in FTD brain; α-synuclein regulates synaptic vesicle endocytosis, not release^42^. This suggests greater synaptic vesicle release in FTD, potentially to compensate for the loss of synapses with increasing neuronal degeneration. Furthermore, we found that NFL and RBFOX3 were decreased, whereas C9ORF72 was increased, in FTD brain, suggesting an increased survival activity in the remaining neurons in FTD brain.

In recent years the interest in the role of lipids in neurodegenerative diseases has come to the fore with increasing realization that lipid changes impact markedly on many neurodegenerative processes. The etiology of FTD in the majority of cases is unknown with a growing number of diverse genes implicated in the disease. The heterogeneous genetic features associated with FTD suggest that multiple pathomechanisms are likely to contribute to the development of FTD. We showed that VLCFA-lipids are significantly increased in FTD brain, likely contributing to neurodegeneration. We therefore suggest that alteration in VLCFA-lipids is a new pathomechanism underlying FTD. Furthermore, we reveal new targets that could be explored for regulating VLCFA-lipids in the brain and therefore reducing neurodegeneration in FTD.

## Methods

### Chemicals and materials

Lipids were extracted using chloroform or methyl-t-butyl ether, methanol and isopropanol (Sigma Aldrich, St. Louis, MO, USA) and ultrapure water (Millipore) as previously described^1^. All solvents used were HPLC grade or higher. Glass pipettes and tubes were used wherever possible and the use of plasticware was minimized during lipid extraction to avoid contamination of samples. Glass tubes and glass transfer pipettes were purchased from Sigma and VWR. Lipid internal standards (ISTDs) were purchased from Avanti Polar Lipids Inc. (Alabaster, AL, USA). These include phosphatidylcholine (19:0), sphingomyelin (12:0), phosphatidylethanolamine (17:0), phosphatidylglycerol (17:0), phosphatidylserine (17:0), phosphatidic acid (17:0), ceramide (d18:1, 12:0), diglyceride (1,3 18:0 d5), cholesteryl ester (19:0), monoglyceride (17:0), triglyceride mix d5 (Avanti Code LM-6000), diglyceride mix d5 (Avanti Code LM-6001), phosphatidylinositol (17:0 14:1), C12 GluCer, C12 sulfatide, C17 ceramide, C17 sphingosine, C17 S1P, C12 C1P, D3 C20 fatty acid, and C12 LacCer. Lipid internal standards were prepared as a mixture at 10 pmol/µl in methyl-tert butyl ether and methanol (MTBE:methanol, 1:1 v/v).

### Human brain tissues

Fresh-frozen post-mortem brain tissue samples were obtained with consent from the Sydney Brain Bank and NSW Brain Tissue Resource Centre. All brain donors underwent standardized assessments in life and standardized neuropathological examination, and met current consensus diagnostic criteria for FTD^43,44^ or no significant neuropathology (controls)^45,46^. Samples from the superior frontal cortex from 10 FTD cases (5 male, 5 female) and 11 controls (5 male, 6 female) were used in this study. The mean age of the two groups were 72.9 ± 13.0 and 79.5 ± 12.1 years, respectively. Ethics approval for the study was from the University of New South Wales Human Research Ethics (approval number: HC15789).

### Patient blood serum

Individuals diagnosed with FTD and healthy controls were recruited at Neuroscience Research Australia in Sydney from FRONTIER, the frontotemporal dementia clinical research group, and from a panel of healthy study volunteers^47^ with no neurological (i.e. no evidence of cognitive or motor impairment) or psychiatric disorders. The study was approved by the University of New South Wales human ethics committee (approval number: HC12573). All methods were carried out in accordance with the relevant guidelines and regulations. Blood samples were obtained following written informed consent from the participant and/or primary carer. All patients underwent a neurological examination, a comprehensive cognitive assessment and structural brain MRI, and met current consensus diagnostic criteria for FTD^48^, as previously described^47^. 40 FTD cases (20 male, 20 female) and 22 controls (9 male, 13 female) were used in this study. The mean age at assessment was 65 ± 8 years for FTD and 71 ± 5 years for controls. Blood samples (9 mL) were collected in tubes (BD Vacutainer SST II Advance Tube #367958), and serum prepared by centrifugation at 3500 rpm for 10 min at 4 °C, which was then aliquoted and stored at − 80 °C until use.

### Lipid extraction

Brain tissue lipid extraction was based on the Matyash method^49^. Briefly, 10 mg of fresh-frozen brain tissues were homogenized in methanol containing 0.01% BHT (300 µl) using Qiagen TissueLyser (3 × 30 s, 30 Hz cycles). The homogenates were transferred to glass tubes, as well as the methanol (430 µl) wash of the beads. MTBE (2.42 ml) was added and the mixture vortexed and incubated for 30 min at room temperature. Water (600 µl) was added and the mixture vortexed and centrifuged at 1000 g for 10 min. The upper phase was transferred to a new glass tube using a glass Pasteur pipette. The lower phase was re-extracted using MTBE/MeOH/water (10:3:2.5). The preparation was dried under nitrogen gas. Dried lipid samples were reconstituted in 100 µl of chloroform/methanol (1:1) and stored at − 80 °C.

Serum lipid extraction was based on the Bligh and Dyer method^50^. Briefly, serum samples were thawed on ice and 80 µl aliquots were transferred into glass tubes. Methanol (600 µl), chloroform (1000 µl) and ultrapure water (500 µl) were sequentially added with vortexing between each addition. Samples were then centrifuged at 3000 rpm for 10 min at room temperature. The lower solvent phase was transferred to a new glass tube using a glass Pasteur pipette. Chloroform (600 µl) was added, vortexed and centrifuged at 3000 rpm for 10 min. The lower phase was collected and transferred into a new glass tube and dried under nitrogen gas. Dried lipid samples were reconstituted in 100 µl of isopropanol/methanol (1:1) and stored at − 80 °C.

### Liquid chromatography/mass spectrometry

Lipid extracts (10 μl) were analyzed using a Q-Exactive Plus Mass Spectrometer coupled to a U3000 UPLC system (ThermoFisher Scientific). Chromatography was performed at 60 °C on a Waters CSH C18 UHPLC column 2.1 × 100 mm, 1.8 μM with VanGuard guard column. Solvent A was 6:4 acetonitrile:water and Solvent B was 1:9 acetonitrile:isopropanol, both with 10 mM ammonium formate and 0.1% formic acid. Lipids were chromatographed according to the method of Castro-Perez et al^51^. Briefly, a 30 min gradient running from 30 to 100% of solvent B was performed, eluting lipids in order of hydrophobicity. Column eluate was directed into the electrospray ionization source of the mass spectrometer where a HESI probe was employed. Source parameters were broadly optimized on a range of lipid standards prior to the analysis. The mass spectrometer was run in data dependent acquisition mode. A survey scan over the mass range 200–1200 at resolution 70 K was followed by 10 data dependent MS/MS scans on the most intense ions in the survey at 15 K resolution. Dynamic exclusion was used to improve the number of ions targeted. Cycle time was approximately 1 s. Samples were run in both positive and negative polarities. The samples were run in a random order (generated using Microsoft Excel). This is important to avoid batch effects/changing instrument performance effects. Data were analyzed in LipidSearch software 4.1.16. Data were searched against the standard Lipidsearch database with all common mammalian lipid classes included. The search results were then grouped according to sample type and aligned for differential analysis. Aligned data (containing lipid identity, retention time, peak area etc.) were exported to Excel software. Relative abundance of lipids was obtained from peak areas normalized to internal standards.

### Protein extraction

Tris-buffered saline (TBS) and SDS-soluble proteins were serially extracted from 100 mg of fresh-frozen brain tissues, as previously described^52^. Briefly, tissues were homogenized in ten volumes of TBS homogenization buffer (20 mM Tris, 150 mM NaCl, pH 7.4, 5 mM EDTA, 0.02% sodium azide) containing protease inhibitor cocktail (Roche) using Qiagen TissueLyser (3 × 30 s, 30 Hz cycles), followed by centrifugation at 100,000 g for 1 h at 4 °C, with supernatant collected as TBS-soluble fraction. The pellet was resuspended in SDS solubilization buffer (TBS homogenization buffer containing 5% SDS) using 3 × 30 s, 30 Hz cycles with TissueLyser, and centrifuged at 100,000 g for 30 min at 25 °C, with supernatant collected as SDS-soluble fraction. Protein concentration was measured using a bicinchoninic acid assay (Pierce BCA Protein Assay Kit) following the manufacturer’s instructions.

### Western blotting and ELISA

Western blotting was carried out as previously described^1^. Protein lysates (10 µg) were heated with sample buffer (3.2% SDS, 32% glycerol, 0.16% bromophenol blue, 100 mM Tris–HCl, pH 6.8, 8% 2-mercaptoethanol). They were then electrophoresed on Criterion Stain-free 4–20% SDS-PAGE gels (Bio-Rad) and transferred onto nitrocellulose membranes at 100 V for 30 min. The membranes were blocked with TBS containing 5% nonfat dry milk and probed overnight at 4 °C with the following antibodies: ELOVL4 (Abcam, ab14925, 1:800), synaptophysin (Invitrogen, MA1-213, 1:1000), RBFOX3 (BioLegend, 834501, 1:2000), NFL (Cell Signaling, 2835S, 1:2000), α-synuclein (BD, 610787, 1:1000), and β-actin (Abcam, ab6276, 1:10,000). They were then washed three times in TBS containing 0.1% Tween 20 and incubated with horseradish peroxidase-conjugated secondary antibodies for 2 h at room temperature. Signals were detected using enhanced chemiluminescence and Gel Doc System (Bio-Rad). The signal intensity was quantified using Image Lab (Bio-Rad) and NIH ImageJ software (v1.45 s). ELOVL4 ELISA was carried out following the manufacturer’s protocol (Abbexa, abx387131).

### RNA extraction and quantitative PCR

RNA was isolated using TRIzol reagent (Invitrogen) following the manufacturer’s protocol as previously described^1^. All procedures were carried out using RNase-free reagents and consumables. One microgram of RNA was reverse transcribed into cDNA using Moloney-murine leukemia virus (M-MLV) reverse transcriptase and random primers (Promega, Madison, Wisconsin, USA) in 20 μl reaction volume. Quantitative PCR (qPCR) assays were carried out using a Mastercycler ep realplex S (Eppendorf, Sydney, Australia) and the fluorescent dye SYBR Green (Bio-Rad), following the manufacturer’s protocol. Briefly, each reaction (20 μl) contained 1 × mastermix, 5 pmol of primers and 1 μl of cDNA template. Amplification was carried out with 40 cycles of 94 °C for 15 s and 60 °C for 1 min. Gene expression was normalized to the geometric mean of three housekeeper genes, GAPDH, β-actin and PPIA. A no-template control was included for each PCR amplification assay. The level of expression for each gene was calculated using the comparative threshold cycle (Ct) value method using the formula 2^−ΔΔCt^ (where ΔΔCt = ΔCt sample—ΔCt reference).

### Gene knockdown and overexpression

SHSY-5Y neuronal cells were cultured in a 12-well plate in Dulbecco's modified Eagle's medium (DMEM) containing 10% fetal calf serum, 1% Glutamax, 0.5% glucose, 100 IU/ml penicillin and 100 μg/ml streptomycin at 37 °C in humidified air containing 5% CO_2_. Knockdown of ELOVL4 was performed using siRNA from Qiagen (Product no. 1027416) and Lipofectamine 2000 as per the manufacturer’s protocol. Scrambled siRNA (Qiagen, Australia) was used as a negative control. Overexpression of ELOVL4 was performed using an ELOVL4 expressing plasmid from Origene (Cat no. RC206248) and Lipofectamine 2000 as per the manufacturer’s protocol. Empty vector, pcDNA (Invitrogen, Australia), was used as a negative control.

### ATP assay

ATP assay was carried out following the manufacturer’s protocol (Abcam, cat. # ab83355) as previously described^1^. Briefly, 50 µl of samples and standards were added to 96-well plates containing the ATP reaction mix (50 µl) and incubated at room temperature in the dark for 30 min. The plates were read using CLARIOstar microplate reader (BMG Labtech) at Ex/Em = 535/587 nm.

### Immunohistochemistry

Formalin fixed, paraffin embedded sections (10 µm) from superior frontal cortex were deparaffinized in xylene and rehydrated through graded ethanol, followed by antigen retrieval with citrate buffer (pH 6.0) using a pressure cooker, and 70% formic acid. Endogenous peroxidase was blocked with hydrogen peroxide, and sections were then blocked with normal horse serum, and incubated with primary antibody against ELOVL4 (Abcam, ab14925, rabbit, 1:50) at 4 °C overnight. Subsequently, sections were incubated with ImmPRESS HRP Horse Anti-Rabbit IgG Polymer Detection Kit (Vector Laboratories, MP-7401), visualized with ImmPACT DAB Substrate, Peroxidase (Vector Laboratories, SK-4105), and counterstained with 0.5% cresyl violet.

### Statistics

Statistical analyses were performed using SPSS Statistics software (IBM, Chicago, Illinois) as previously described^1^. Multivariate analyses (general linear model) covarying for age and sex were used to determine differences in lipid and protein levels in the FTD and control groups with posthoc statistical significance set at *p* < 0.05. Pearson’s correlations were used to determine if changes in measurements were associated with each other with statistical significance set at *p* < 0.05. In vitro cell studies were conducted in n = 6 replicates and the whole experiment repeated at least twice as indicated in the figure legends, and significance was determined using the Student’s *t*-test and *P* < 0.05 considered significant.

## Supplementary Information

Supplementary Information.
