# Supplementary material for: Increased VLCFA-lipids and ELOVL4 underlie neurodegeneration in frontotemporal dementia
Source: Sci Rep. 2021 Nov 1;11:21348. doi: 10.1038/s41598-021-00870-x (PMC8560873; doi:10.1038/s41598-021-00870-x)

Suppl Fig. 1

ELOVL4

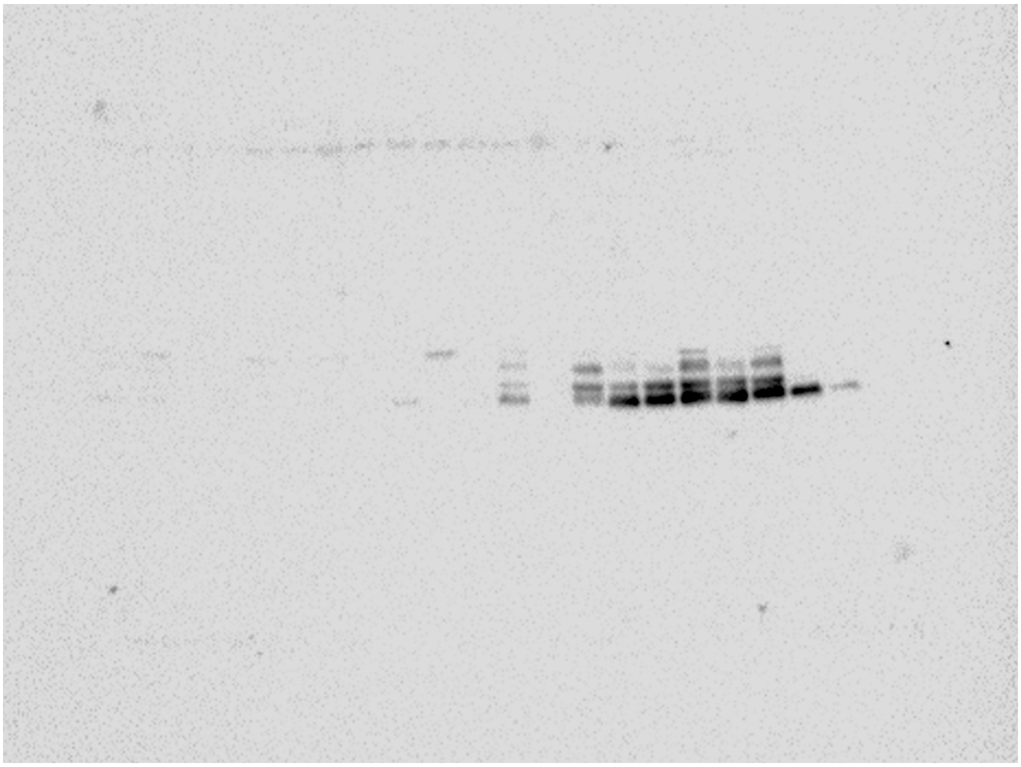

β-actin

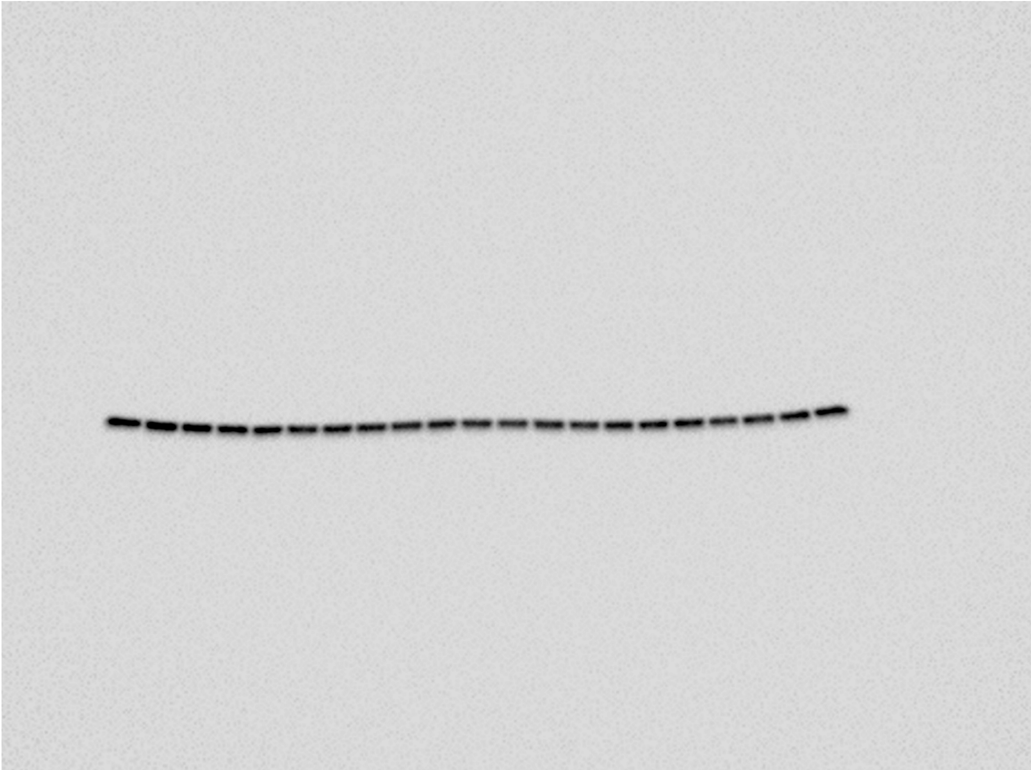

Suppl Fig. 2

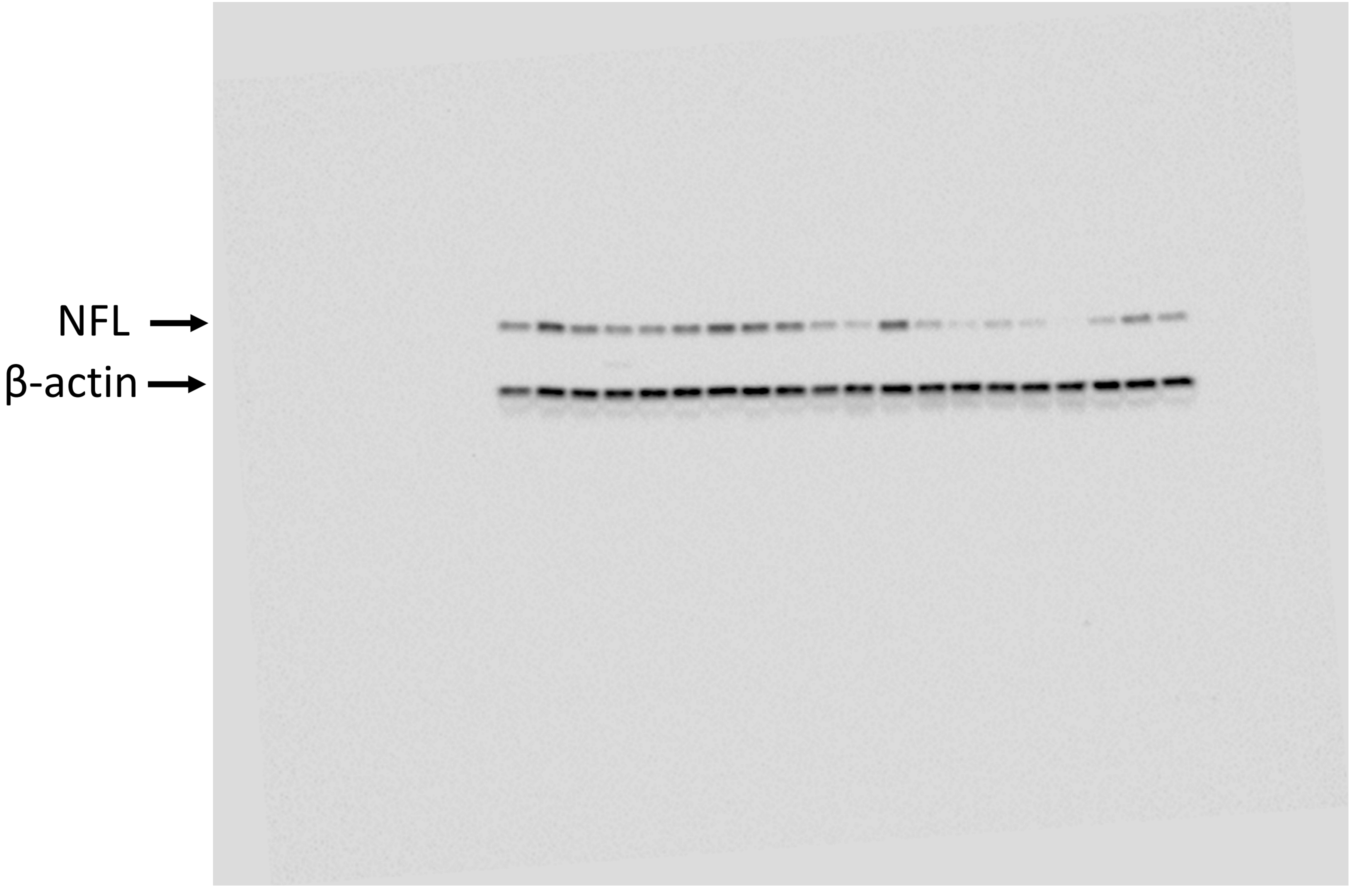

Suppl Fig. 3

RBFOX3

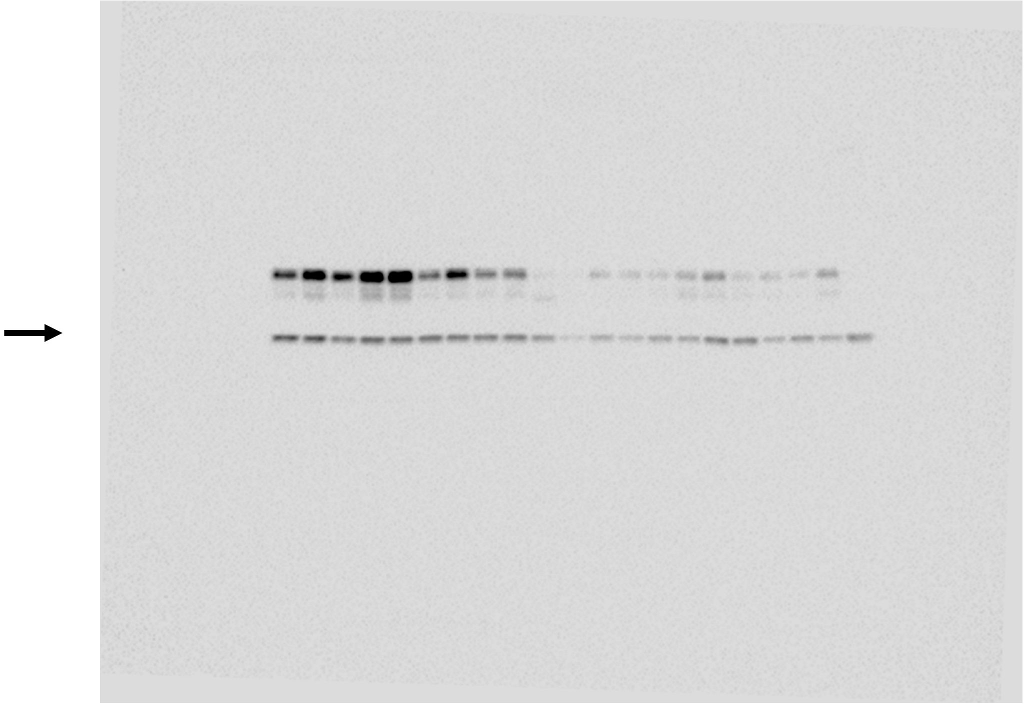

β-actin

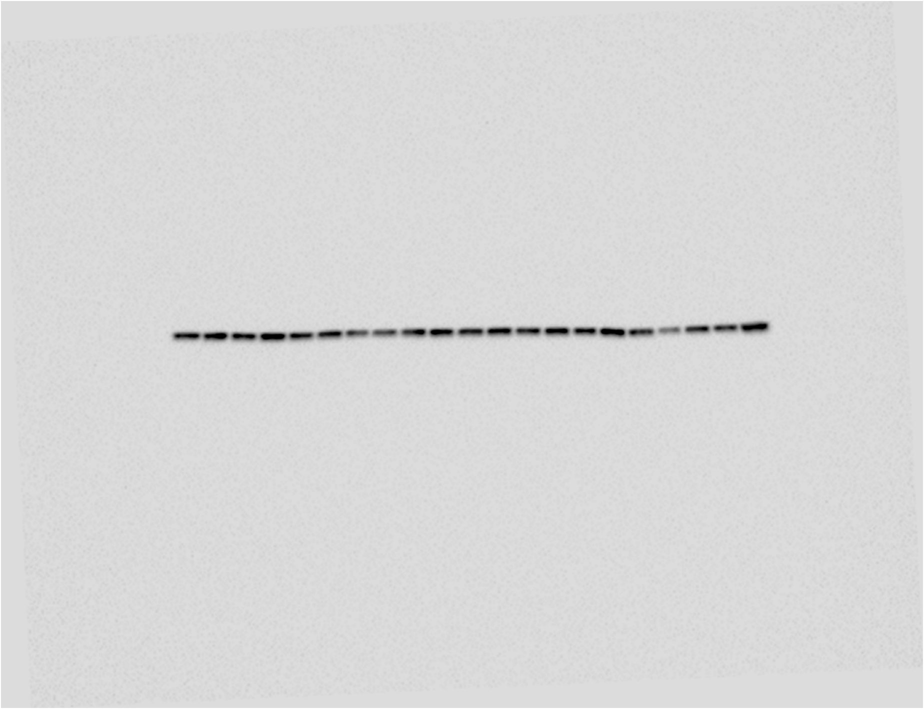

Suppl Fig. 4

C9ORF72

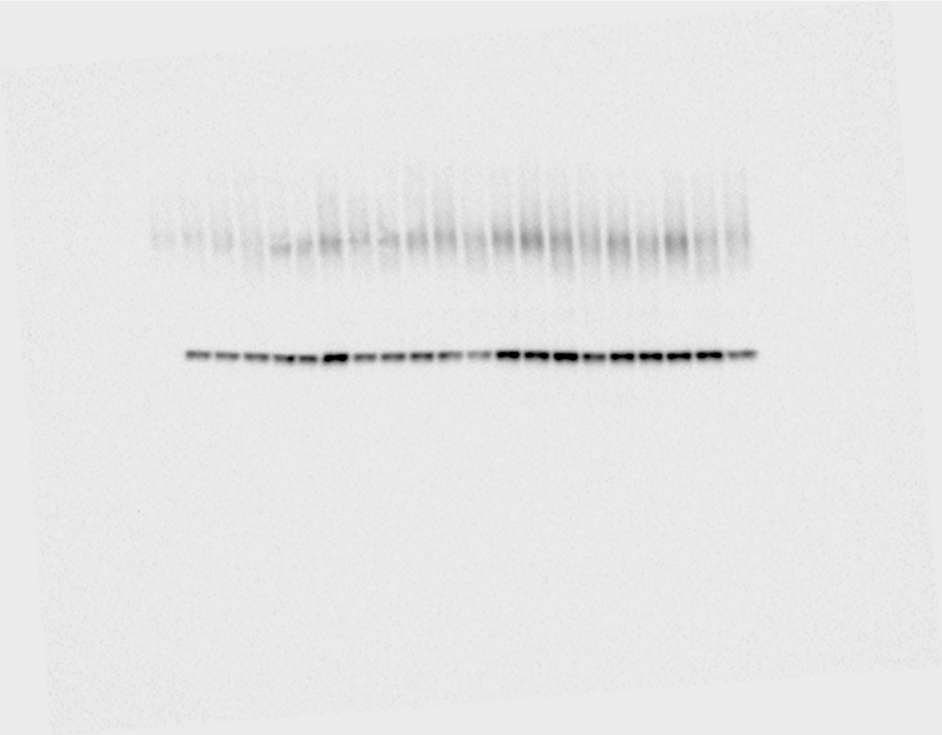

β-actin

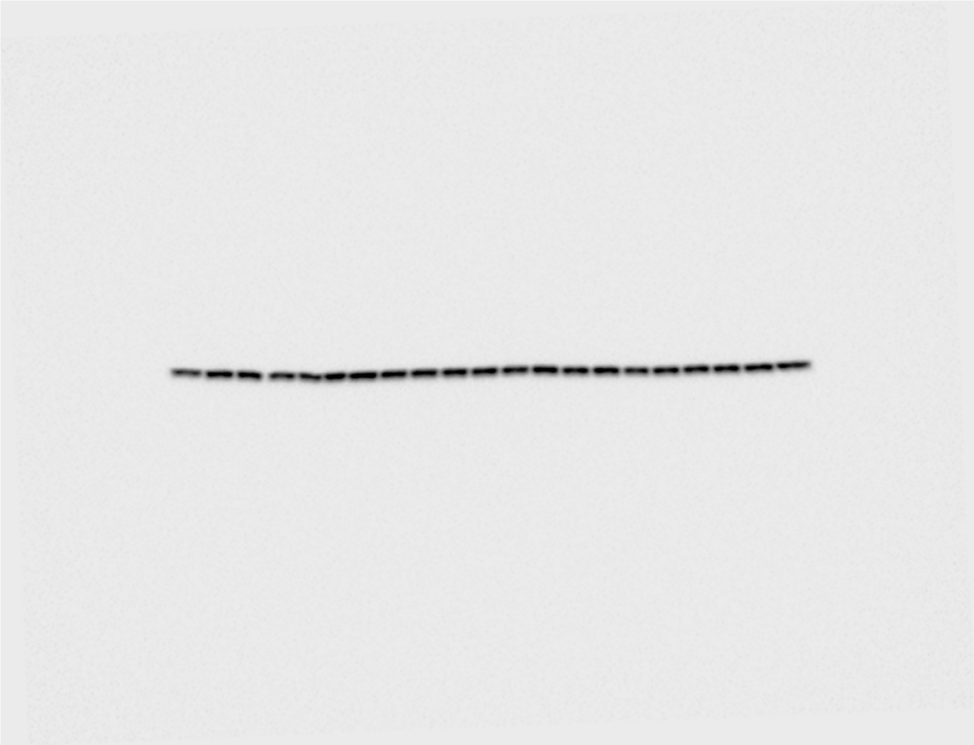

Suppl Fig. 5

SYP

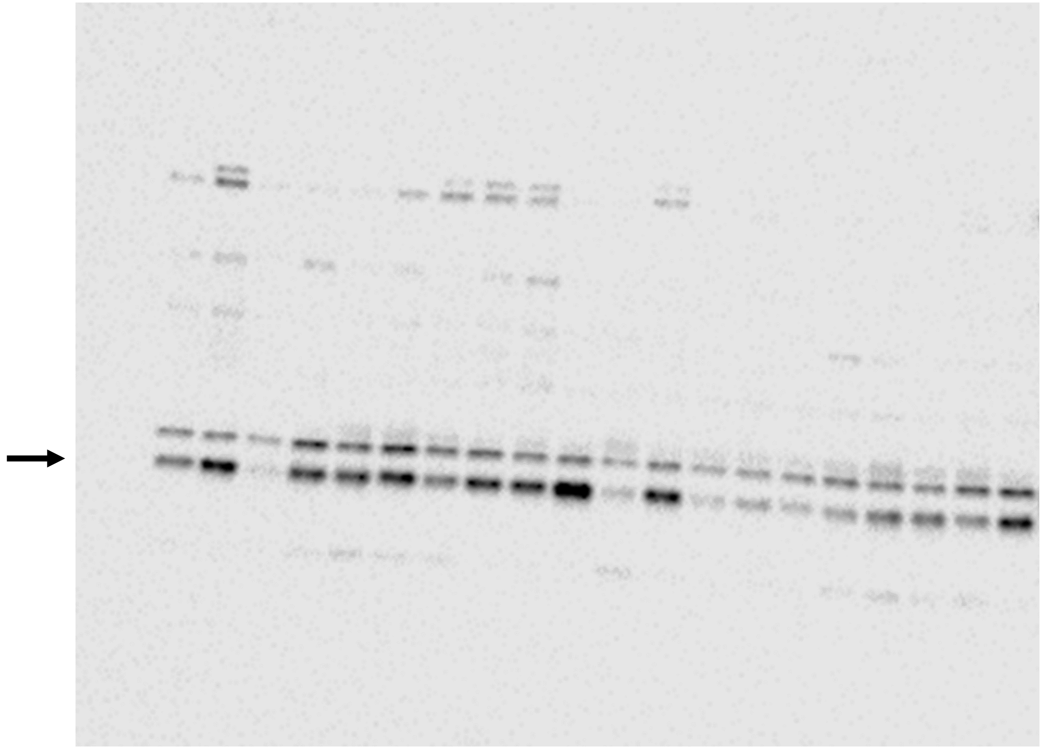

β-actin

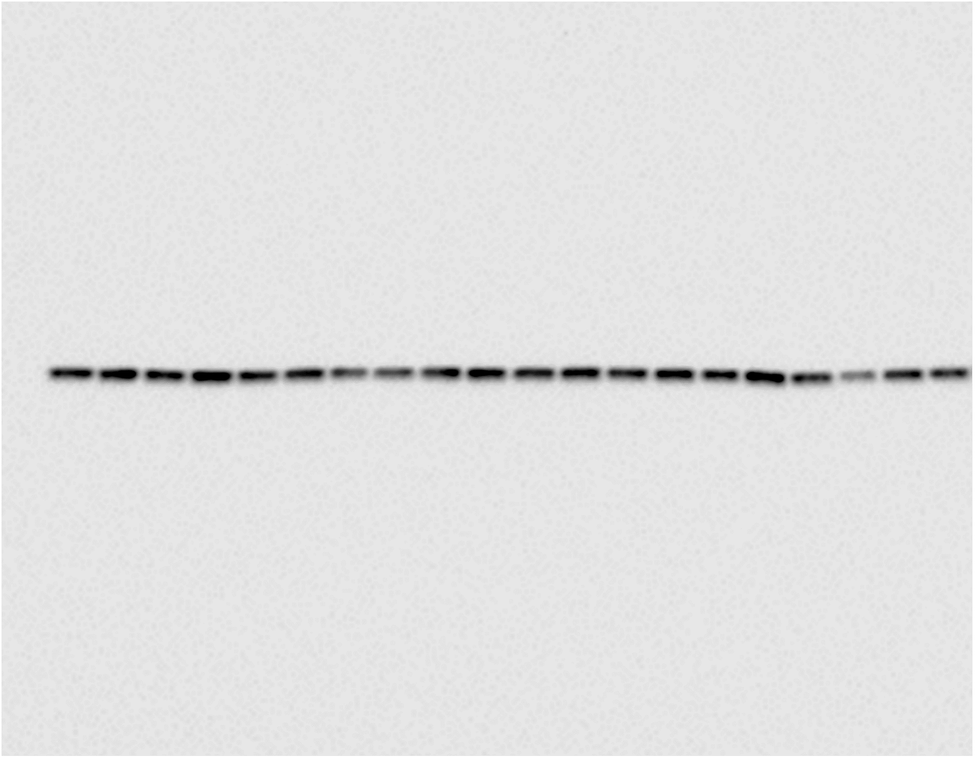

Suppl Fig. 6

α-Syn

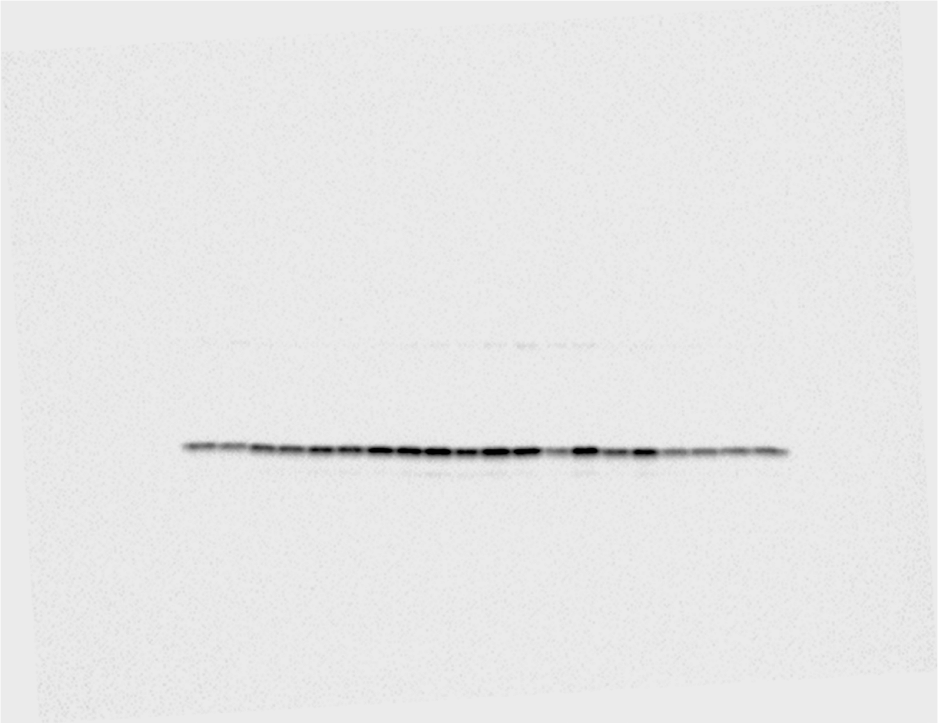

β-actin

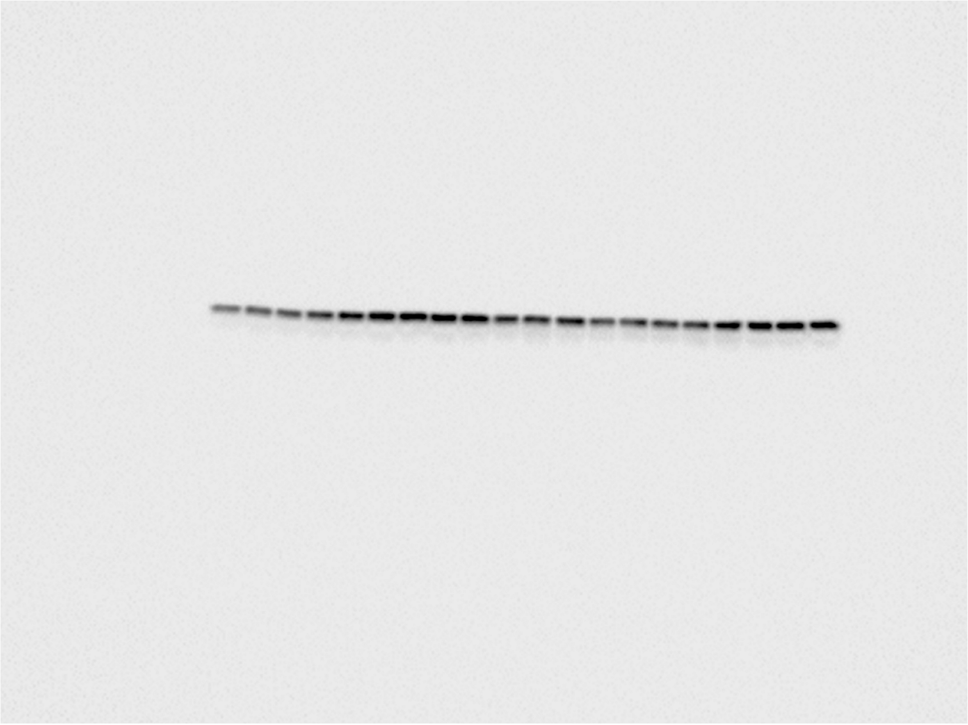

Suppl Fig. 7

ABCD1

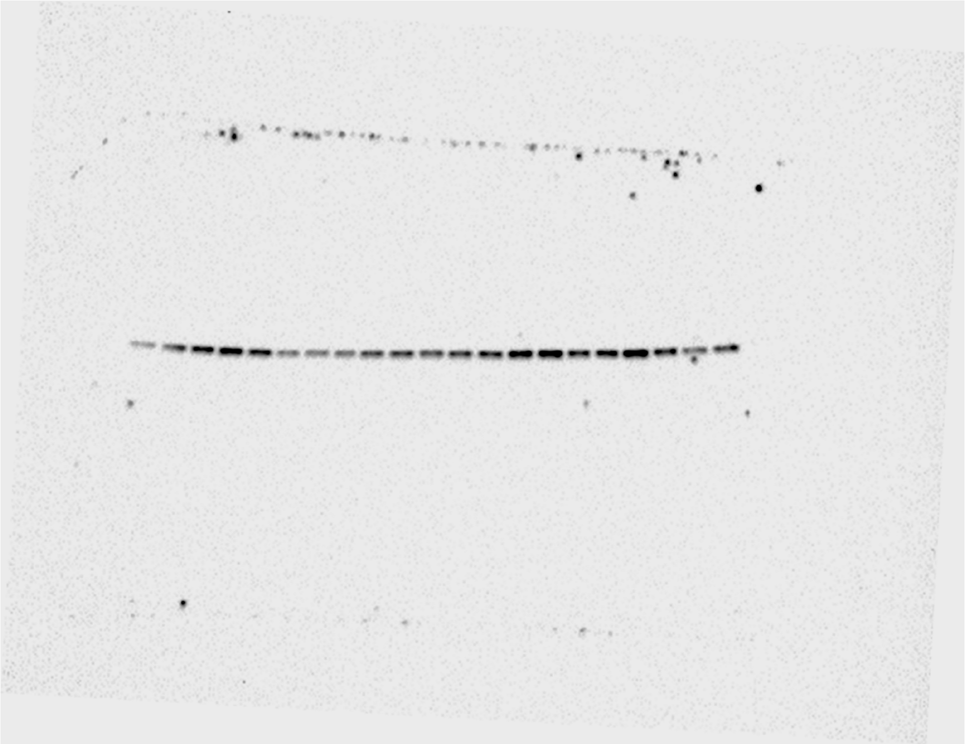

β-actin

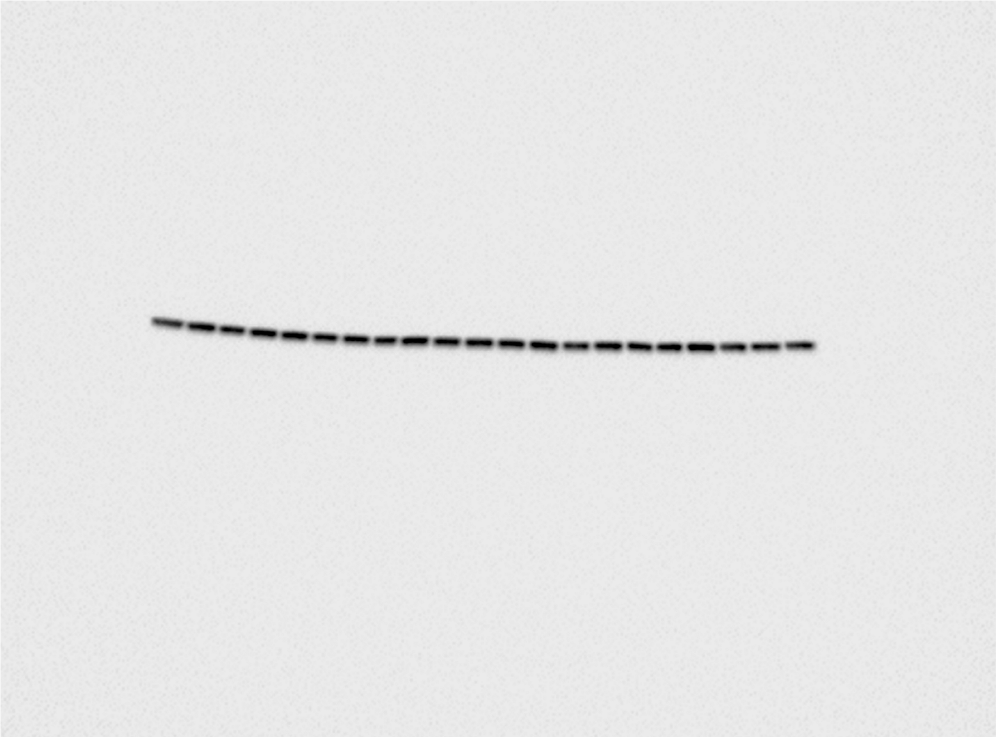

Supplement: Supplementary file 1 — Supplementary Information. [file 41598_2021_870_MOESM1_ESM.pdf]
